# The role of circulating tumor DNA in melanomas of the uveal tract

**DOI:** 10.3389/fimmu.2024.1509968

**Published:** 2024-12-04

**Authors:** Mohammed Zeeshan Zameer, Eric Jou, Mark Middleton

**Affiliations:** ^1^ Department of Oncology, Medical Sciences Division, University of Oxford, Oxford, United Kingdom; ^2^ Kellogg College, University of Oxford, Oxford, United Kingdom

**Keywords:** uveal melanoma, circulating tumor DNA (ctDNA), molecular residual disease, minimal residual disease, liquid biopsy, biomarker

## Abstract

Melanoma of the uveal tract or uveal melanoma (UM) originates from melanocytes of the eye and is the most common intraocular malignancy in adults. Despite considerable advances in diagnostic procedures and treatments, prognosis remains poor in those with advanced disease. Accordingly, although current treatments have an excellent local disease control rate, approximately 50% of patients develop metastatic relapse within 10 years. The high risk for metastatic disease with a variable and often long latency period is thought to be due to early spread of cancer cells disseminating into organs such as the liver, followed by a period of dormancy, before the eventual emergence of radiologically measurable disease. Early detection of disease relapse or metastasis is therefore crucial to allow timely treatment and ultimately improve patient outcome. Recently, advances in minimally-invasive liquid biopsy techniques and biomarkers such as circulating tumor DNA (ctDNA) have demonstrated potential to transform the field of cancer care by aiding diagnosis, prognosis and monitoring of various cancer types. UM is particularly suitable for ctDNA-based approaches due to the relatively well-characterized spectrum of genetic mutations, along with the inherent difficulties and risks associated with getting sufficient tumor samples via traditional biopsy methods. Key potential advantage of ctDNA are the detection of molecular residual disease (MRD) in patients post definitive treatment, and in the early identification of metastasis. This is particularly relevant contemporarily with the recent demonstration of tebentafusp improving survival in metastatic UM patients, and opens avenues for further research to investigate the potential utilization of tebentafusp combined with ctDNA-based strategies in adjuvant settings and early intervention for MRD. The present review illustrates the current understanding of ctDNA-based strategies in UM, discusses the potential clinical applications, explores the potential of utilizing ctDNA in UM MRD in the context of an ongoing clinical trial, and highlights the challenges that need to be overcome prior to routine clinical implementation.

## Introduction

Melanomas of the uveal tract or uveal melanoma (UM) are rare and highly aggressive malignancies that arise from eye melanocytes, and can be found in the iris, ciliary body and choroid, the latter consisting of the vast majority (90%) of total cases (1). UM is the most common intraocular malignancy in adults with an estimated worldwide incidence of 7000 cases annually (2), and despite considerable advances in diagnosis and treatment modalities in recent years, prognosis remains poor in particular for metastatic disease (3). Accordingly, development of distant metastasis is seen in up to 50% of UM patients, with the liver being the most common site followed by lung and bone (4, 5). Time to distant metastasis is highly variable with some patients developing metastasis 10 years after initial therapy (6), and median survival after detection of metastasis is poor ranging from 3.9 to 9 months (7). Until recently, systemic treatments such as immune checkpoint inhibitors or chemotherapy have been largely disappointing in UM, and the recent advent of tebentafusp demonstrated unprecedented efficacy in improving survival (8). There is therefore a dire need of strategies to improve and facilitate early detection of disease relapse or metastasis after definitive treatment, which will provide lead time for early therapeutic intervention.

In recent years, there are growing efforts aimed at developing and utilizing liquid biopsy-based strategies against solid cancers due to the several perceived benefits such as being less invasive with fewer side effects, provide a better assessment of tumor heterogeneity, and can be repeatedly taken longitudinally to monitor clinical course (9–11). In particular, circulating tumor DNA (ctDNA)-based approaches gained traction due to the relatively higher stability of DNA compared to other molecules such as RNA, and being less fragile than circulating tumor cells (CTCs) (12–14). Proposed clinical applications of ctDNA include aid for cancer diagnosis, assess for molecular residual disease (MRD), provide prognosis value and risk of metastasis, early detection of disease relapse or metastasis, analyze tumor heterogeneity and spectrum of tumor genetic alterations, help treatment selection and assessing treatment response. UM as a cancer type is particularly amenable to ctDNA-based strategies, due to the relative difficulty and the associated risks of serious complications such as blindness when acquiring primary tumor samples, and its well-characterized mutational landscape which allows for tumor-agnostic approaches (15–17). Furthermore, the eye is notorious for having a poor lymphatic supply hence likely spreads through the hematogenous route (18), and provides rationale for detecting ctDNA in the blood.

Currently, the use of ctDNA in UM is largely limited to research settings. In this review, existing evidence of ctDNA-based strategies in solid tumors are briefly illustrated, followed by a thorough discussion on the contemporary evidence and potential of utilizing ctDNA in UM patient care. The high risk of developing metastatic disease with variable latency periods in UM patients suggest that cancer spread may be an early phenomenon that long precede clinical detection of metastasis (19). The potential use of ctDNA in detecting MRD after definitive treatment and early identification of metastasis is therefore crucial and is also highlighted in the context of a recent ongoing trial TebeMRD (EudraCT number: 2019-003946-34) that attempts to address some of these key questions. Current technological limitations on ctDNA detection and the challenges in clinical implementation are also examined.

## Overview of ctDNA and utilities in cancer management

There are increasing efforts over recent years to develop and utilize liquid biopsy-based strategies in the care of patients with solid tumors due to the many perceived benefits. Compared to conventional surgical biopsies, liquid biopsies are less invasive, impose a lower risk of iatrogenic dissemination of the primary tumor, and the procedures are in most cases well-tolerated by patients (20). Furthermore, liquid biopsies may provide additional information compared to conventional methods on spatial and temporal tumor heterogeneity, which are major drivers of cancer therapeutic resistance (21). During carcinogenesis, tumors increase in heterogeneity as the disease progresses, resulting in the development of genetically distinct subpopulations both within the primary tumor and at distant metastatic sites (22). Liquid biopsies have the potential to capture products derived from a broader range of tumor subpopulations across multiple sites, and can be taken repeatedly in a longitudinal manner throughout the treatment course of the patient. Accordingly, potential clinical applications of liquid biopsies include earlier cancer diagnosis, assess for molecular or minimal residual disease through detecting molecules (e.g. ctDNA) or cancer cells respectively, monitor for disease relapse or metastasis, characterize tumor genetic alterations, provide prognostic information, assess risk of metastasis, analyze tumor heterogeneity, inform treatment selection, and determine treatment response.

Of the range of liquid biopsy analytes, which include ctDNA, CTCs, circulating tumor RNA (ctRNA), circulating microRNA (miRNA) and extracellular vesicles, ctDNA have clear advantages as DNA is more stable than RNA, and a plethora of more robust and standardized methods for detecting and analyzing ctDNA are available compared to the more fragile CTCs (12–14). Several techniques have been developed to detect ctDNAs over the past decade with varying sensitivities and specificities, with the most prominent being methods based on digital droplet polymerase chain reaction (ddPCR) and next-generation sequencing (NGS) (**Table 1**) (33). Techniques based on ddPCR are highly sensitive and can be used to assess for various types of genetic alterations such as target mutations and somatic copy number alterations (SCNA), however are limited to known genetic aberrations which needs to be defined prior to use (34). On the other hand, NGS-based methods do not require prior knowledge of the sample DNA sequence, can be utilized to sequence the whole genome or specific target regions, and allow for exploratory analyses to identify unknown mutations (35). Conversely, NGS-based methods require a higher level of bioinformatics analysis and have a longer turn-around time than ddPCR, hence may be less suitable in certain time-sensitive clinical scenarios (36, 37). Strategies to improve ctDNA detection are continuously being sought for to increase utility, reduce false positive rates, and facilitate clinical implication. For example, recently a pipeline named Integration of Variant Reads (INVAR) combining custom error-suppression and signal-enrichment approaches was developed, and was able to further improve the limit of ctDNA detection (38).

**Table 1:** Current ctDNA detection technologies and the respective advantages and disadvantages (23–32).

Technology	Advantages	Disadvantages
ddPCR	High sensitivity and specificity, cost-effective, quantitative	Limited to known mutations, lower throughput compared to NGS
NGS	Broad scope, high throughput, no prior knowledge of mutations required.	Costly, lower sensitivity, complex interpretation
Bi-PAP PCR	High sensitivity, quantitative	Lower throughput
BEAMing	High sensitivity, quantitative	Labor-intensive, high cost
CAPP-Seq	High sensitivity, customizable	Complex workflow, high cost
Safe-SeqS	High accuracy, sensitive	High cost, requires complex bioinformatics
Hybrid capture-based NGS	Detects broad spectrum of genetic alterations, customizable	Expensive, complex computational analysis

NGS, next generation sequencing; ddPCR, droplet digital polymerase chain reaction; Bi-PAP, bidirectional pyrophosphorolysis-activated polymerization; BEAMing, beads emulsion amplification magnetics digital PCR; CAPP-Seq, cancer personalized profiling by deep sequencing; Safe-SeqS, safe-sequencing system.

Strategies to utilize ctDNA in patient care can be categorized into either tumor-informed or tumor-agnostic approaches. The tumor-informed approach is dependent on prior knowledge acquired through genomic profiling of the primary tumor tissue, while the tumor-agnostic strategy is performed independently of the genomic information from the primary tumor. Improved sensitivity and specificity can be achieved via the tumor-informed approach, for example through filtering out non-relevant mutations (39), yet are limited by having access to primary tumor tissues which may not always be logistically or clinically feasible. Where primary tumor samples are limited for example due to the increasing use of neoadjuvant therapy or small initial tumors, or in cases where invasive acquisition of primary tumor samples are challenging or associated with risks of serious complications, a tumor-agnostic approach has the potential to provide invaluable information about the cancer and help guide clinical management. Accordingly, the decision to determine the most suitable approach will depend on the cancer type and the clinical features of the particular patient.

Emerging data in recent years demonstrate the potential clinical utilities of ctDNA in cancer patient care. Mechanistically, circulating free DNA (cfDNA) are released during cell death processes such as apoptosis, necrosis, ferroptosis, oncosis, NETosis and pyroptosis, and also actively by live cells through vesicular pathways via autophagy or exosomes (40–42). A greater proportion of cfDNA is occupied by ctDNA in advanced stage cancer compared to early disease, with figures ranging from 0.003% to 95% of total cfDNA (40). Accordingly, higher ctDNA levels have been shown to correlate with disease stage in colorectal cancer (CRC) and tumor burden in cutaneous melanoma (43, 44). Furthermore, ctDNA-based approaches have been shown to predict disease recurrence after curative surgery in stage II CRC patients (45), effectively analyze mutation status in non-small cell lung cancer (NSCLC) patients allowing identification of candidates for targeted therapies (46), and provide prognostic information in cutaneous melanoma patients (47). These studies highlight the versatility and the broad range of potential clinical applications of ctDNA. In the case of UM, currently the clinical utilities of ctDNA-based approaches are still in early stages, and are actively being investigated.

## Rationale of ctDNA-based approaches in UM

Amongst solid tumors, UM is a prime candidate for ctDNA-based approaches due to its unique characteristics, pathogenesis and clinical course, and the potential benefits of utilizing ctDNA-based strategies are multi-fold. Firstly, the quantity of primary UM tumor tissue available for molecular characterization is often limited, in particular after eye-sparing irradiation treatments, and there is a growing preference contemporarily towards conservative therapies aimed at eye preservation (48, 49). Furthermore, early diagnosis of UM is imperative for better patient outcome (50), yet comprehensive prognostic assessment of early stage small tumors can be challenging due to the scarcity of primary cancer tissue for molecular analysis. Strategies that utilize ctDNA may overcome these challenges by providing additional cancer-derived material that are relatively easily accessible for analyses. Secondly, the unique anatomical relations of UM allow additional sources for liquid biopsies in addition to blood, including vitreous and aqueous humor which have been shown to harbor ctDNA (51, 52). Thirdly, the eye has poorly developed lymphatic drainage systems and UM is largely thought to metastasize through the blood route (18), providing rationale for blood-based ctDNA detection strategies in the screening and early detection of metastasis. Upon initial diagnosis of UM, just under 4% of patients have radiological evidence of metastatic disease, however this rises to 50% within 10 years of diagnosis, highlighting the need of robust surveillance methods for metastasis performed in sufficient frequencies in order to facilitate early detection (53, 54). Finally, techniques with high sensitivities to detect ctDNA such as ddPCR require known mutation targets. UM is also ideal in this regard as a panel of four mutually-exclusive mutated genes with known mutational hotspots, namely *GNAQ*, *GNA11*, *PLCB4* and *CYSLTR2*, which encodes for guanine nucleotide-binding protein G(q) subunit α, guanine nucleotide-binding protein subunit α11, phospholipase C β4, and cysteinyl leukotriene receptor 2 respectively, collectively accounts for almost all UM cases thereby allowing tumor agnostic approaches (15–17). Coupled with the fact that liquid biopsies impose significantly less risk of serious complications such as vision loss compared to intraocular tumor biopsies, the envisaged benefits of ctDNA-based approaches in UM is potentially multi-fold and should be validated in clinical trials.

## UM pathogenesis and ctDNA gene targets

Advances in the understanding of UM molecular pathogenesis is crucial to identify suitable gene targets and facilitate adoption of ctDNA-based strategies in UM clinical care. Approximately 80-90% of UMs have activating mutations in either *GNAQ* or *GNA11* in a mutually exclusive pattern, leading to constitutively active G protein-coupled receptor (GPCR) signaling (15). Gq and G11 signals through activating phospholipase C-β (PLCβ), which converts phosphatidylinositol bisphosphate (PIP2) into inositol triphosphate (IP3) and diacylglycerol (DAG). IP3 induces calcium release from the endoplasmic reticulum which primes protein kinase C (PKC), allowing the latter to bind to DAG resulting in PKC activation (55). PKC induces the well-established cancer-promoting mitogen-activated protein kinase (MAPK) signaling pathway through activating Ras which in turn simulates Raf and downstream MAPKs (56, 57). Mechanistically, MAPK signaling is one of the most potent pathways in promoting cell proliferation, and in addition exerts anti-apoptotic effects and facilitates cancer invasion through promoting epithelial to mesenchymal transition (EMT) (58), all of which are hallmarks of cancer and is a prime target for cancer therapies (59, 60). Of the remaining UM without mutations in *GNAQ* or *GNA11*, activation of the PKC-MAPK pathway is also observed in the majority of cases through activating mutations in *CYSLTR2* or *PLCB4* (17, 61), which encodes for the GPCR cysteinyl-leukotriene receptor 2 (CysLTR2) and PLC-β4 respectively. Importantly, UM lacks the mutations commonly found in cutaneous melanoma such as *BRAF* or *NRAS*, making them unsuitable for targeted approach against these with the current range of inhibitors available clinically (62). Given the convergence of *GNAQ*, *GNA11*, *CYSLTR2* and *PLCB4* mutations on PKC activation and downstream MAPK signaling, PKC serves as an attractive therapeutic target. Accordingly, PKC inhibitors are currently being investigated in UM clinical trials showing promising results in terms of safety and efficacy (56).

UM is distinct to cutaneous melanoma with the former having a low mutational rate (17), which is consistent with the disappointing lack of efficacy of immune checkpoint inhibitors in UM compared to cutaneous melanoma (63). Instead, UM has a unique profile of chromosomal aberrations and gene mutations with known mutational hotspots, rendering them amenable to ctDNA-based approaches as knowledge on tumor mutational profiles increases ctDNA detection sensitivity. Genetic aberrations in UM can be categorized into cancer oncogenesis driver genes and prognostic genes. Known driver mutations include the aforementioned *GNAQ*, *GNA11*, *CYSLTR2* and *PLCB4*, which are mutually exclusive and found in 24.2 to 53.3%, 24.2 to 60%, 4%, and 2.5% of UM respectively, together accounting for almost all UM cases (15). *GNAQ* and *GNA11* are paralogous genes found on chromosome 9q21.2 and chromosome 19p13.3 respectively with a 90% sequence homology, and share mutational hotspots. The most common mutations are activating missense variants at Q209 in exon 5, followed by arginine R183 in exon 4 (15). Single base substitutions at codon 209 replacing glutamine with leucine or proline results in complete abrogation of GTPase activity leading to aberrant G protein activation, and similarly R183, while less essential than Q209, also has important contributory roles to GTP hydrolysis (64). Hotspot mutations have also been identified for *CYSLTR2* and *PLCB4*, which are found to affect L129 and D630 respectively (16, 65). Although useful for molecular characterization and diagnosis, most studies to date are in agreement that the driver genes *GNAQ* and *GNA11* do not provide prognostic value, while it remains unclear for *CYSLTR2* and *PLCB4* as currently there are few available studies that interrogated their prognostic role (66, 67). Therefore, ctDNA methods that look for mutations in these genes are largely aimed at diagnostic or clinical monitoring purposes such as response to treatment, instead of prognostication or predicting metastatic risk.

On the other hand, an array of genetic aberrations that provide prognostic value in UM have been identified, including mutations in *BAP1*, *SF3B1* and *EIF1AX*, and chromosomal SCNA such as monosomy 3, 8q amplification, 6p gain and deletion of 1p, 8p and 16q (68–72). Mutations in genes encoding BRCA1-associated protein 1 (*BAP1*), splicing factor 3 subunit B1 (*SF3B1*) and eukaryotic translation initiation factor 1A (*EIF1AX*) occur in a mutually exclusive pattern, and are found in 45%, 24% and 17% of UM respectively (73). BAP1 is a deubiquitinating enzyme involved in chromatin remodeling with established tumor suppressor activity (74), and biallelic loss of *BAP1* function typically occurs through loss of chromosome 3 (which contains *BAP1*) and loss of function mutations in the other allele (75). Unlike oncogenes such as *GNAQ* and *GNA11* which require specific activating mutations resulting in mutational hotspots, as a tumor suppressor loss of function mutations in *BAP1* lack a clearly defined pattern, hence sequencing techniques are required for ctDNA analyses to capture the wide range of mutations in *BAP1*. On the other hand, *EIF1AX* and *SF3B1* have oncogenic function (76, 77) with known mutational hotspots in exon 1 and 2 for *EIF1AX*, and codons R625, K666 and K700 for *SF3B1* (78–80). UM can be classified based on gene expression profile (GEP) into class 1 with low metastatic risk and class 2 with high risks of metastasis and poor survival (81). *BAP1* mutations are found to be associated with class 2 gene expression profile (GEP), and has been found to be amongst the strongest predictors of metastasis (RR = 10.6, 95% CI 3.4-33.5) and melanoma-specific mortality (RR = 9.0, 95% CI 2.8-29.2) after excluding GEP class (73). BAP1 mutations predict early metastasis risk, while presence of *EIF1AX* mutations correlates with GEP class 1 and low metastatic risk, and SF3B1 mutations are associated with late metastasis (73, 82). More recently through comprehensive multiplatform analysis largely based on somatic alterations and gene expression profiles, The Cancer Genome Atlas (TCGA) classification system identified four molecularly distinct subsets of UM with important prognostication value (**Table 2**) (83, 84). Cluster A or cluster 1 UM are characterized by mutations in *EIF1AX* and gain of chromosome 6 or neutral SCNA profiles, while cluster B or cluster 2 harbors SF3B1 mutations, gain of chromosome 6p, loss of chromosome 6q, or gain of chromosome 8q. Cluster C (cluster 3) and cluster D (cluster 4) are depicted by having mutations in *BAP1*, gain of chromosome 8q, and loss of chromosomes 1p, 3, 6q, 8p, and are subdivided into cluster 3 and cluster 4 based on level of 8q gains which are higher in cluster 4. In a landmark study where 658 UM patients were categorized based on the TCGA classification system (84), cluster A was found to be associated with good prognosis with a 5-year metastasis risk from diagnosis at 4%, compared to 20% for cluster B, 33% for cluster C and 63% for cluster D. 5-year hazard ratio (HR) for metastasis were 4.1 (P = 0.01), 10.1 (P < 0.001), and 30.0 (P < 0.001) respectively for cluster B, C and D when compared to cluster A. Cluster D is also associated with higher mortality when compared to cluster A with a 5-year HR for death of 13.7 (P < 0.001). The overall estimated risk of metastasis was 3%, 10%, 25% and 41% for metastasis (P < 0.001), and 1% *vs* 0% *vs* 3% *vs* 9% for death (P < 0.001), in cluster A, B, C, and D respectively. These findings highlight the relevant genetic aberrations that can be interrogated in UM patients to stratify metastatic risk and determine prognosis, and opens avenues for ctDNA-based approaches in UM molecular prognostication and patient stratification.

**Table 2:** Potential genetic aberration targets for ctDNA analysis with prognostication value in relation to TCGA clusters.

TCGA cluster	Genetic aberrations	Prognosis
Cluster A (cluster 1)	• *EIF1AX* mutations • Chromosome 6 gain or neutral SCNA	• Good prognosis • GEP class 1 • 5 year metastasis risk of 4%.
Cluster B (cluster 2)	• *SF3B1* mutations • Chromosome 6p gain • Chromosome 8q gain (low) • Chromosome 6q loss	• Intermediate prognosis • GEP class 1 • 5 year metastasis risk of 20%.
Cluster C (cluster 3)	• *BAP1* mutations • Chromosome 1p, 3, 6q and/or 8p loss • Chromosome 8q gain (intermediate)	• Poor prognosis • GEP class 2 • 5 year metastasis risk of 33%.
Cluster D (cluster 4)	• *BAP1* mutations • Chromosome 1p, 3, 6q and/or 8p loss • Chromosome 8q gain (high)	• Very poor prognosis • GEP class 2 • 5 year metastasis risk of 63%.

TCGA, The Cancer Genome Atlas; GEP class, Gene Expression Profile class; SCNA, somatic copy number alterations; *EIF1AX*, eukaryotic translation initiation factor 1A; *SF3B1*, splicing factor 3 subunit B1; *BAP1*, BRCA1-associated protein 1.

Methods that utilise ctDNA may facilitate prognostication through the detection of an array of uveal melanoma (UM) genetic aberrations and guide subsequent management.

## Clinical utilities of ctDNA in UM

The potential clinical benefits of incorporating ctDNA-based approaches in UM management are multi-fold, including but not limited to UM diagnosis, disease monitoring, assess treatment response, early detection of metastasis, molecular prognostication, identifying patients for targeted therapy, and allowing timely treatment through early diagnosis of disease recurrence or metastasis.

Early diagnosis of UM is crucial to improve patient outcomes. In a retrospective study of 8033 eyes, increased UM primary tumor diameter or thickness is associated with increased metastatic risk (50). Furthermore, the risk of metastasis within 10 years increases as depth increases, at 6% for UM with 0-1.0 mm thickness compared to 51% for those >10.0 mm, highlighting the importance of rapid and accurate diagnosis of UM to allow early treatment. Currently, UM diagnosis is largely reliant on ophthalmologists experienced in ocular tumors to differentiate between benign naevi and malignant UM based on clinical examination findings and various imaging modalities such as fluorescein angiography and ocular echography. The potential role of ctDNA in the initial diagnosis of UM would likely be complementary, and may be particularly useful in more challenging cases of indeterminate choroidal melanocytic lesions where diagnosis is uncertain, or in cases where tumors are small and less amenable to conventional biopsy methods. Furthermore, conventional intraocular biopsies are associated with risks, and it can be difficult to ascertain the risks and benefits of intraocular biopsies in uncertain cases, while minimizing misdiagnosis of UM as naevus or macular degeneration. Insufficient material for diagnosis was also suggested to occur in up to 22% of cases with fine-needle aspiration biopsies (85). Strategies detecting ctDNA through liquid biopsies may overcome these challenges and provide vital diagnostic information, in particular when targeted towards identifying driver gene mutations in *GNAQ*, *GNA11*, *CYSLTR2* and *PLCB4*, which collectively occurs in almost all UM cases, and also genes and SCNAs associated with metastatic risk such as *BAP1*, *EIF1AX*, *SF3B1* and chromosomal alterations. In a study utilizing ddPCR to detect mutations in the driver genes *GNAQ*, *GNA11*, *CYSLTR2* and *PLCB4*, patients with UM were found to have higher levels of ctDNA when compared to patients with naevi, and ctDNA levels correlated strongly with malignancy (86), presumably due to higher cell turnover in malignancy. These promising findings indicate that ctDNA analysis of driver genes have the potential to determine UM from benign naevi, and may also be useful in following-up and monitoring individuals with choroidal naevi which require regular monitoring due to the risk of transformation (87). On the other hand, another important factor to consider is ctDNA detection rate in primary UM, which ranges from only 2% to 26% across different studies, though this increases significantly in patients with recurrent disease or metastasis (up to 94%) (23, 25, 26, 88). Therefore, a negative ctDNA result cannot rule out a diagnosis of primary UM. Overall, whilst the use of ctDNA to resolve diagnostic uncertainty has potential, at present the existing evidence do not provide sufficient confidence in the techniques to allow reliance on ctDNA-based approaches as a diagnostic aid during initial diagnosis. Further scientific advances on this front are necessary to achieve the robustness required for clinical utilization.

In addition to initial UM diagnosis, ctDNA-based approaches may also be used to provide prognostication and predict metastatic risk in UM patients through determining gene mutations and SCNAs. Biallelic inactivation of the *BAP1* gene is associated with high risks of metastasis and poor patient prognosis, and as mentioned typically occurs through loss of one allele due to monosomy chromosome 3 coupled with loss of function mutations in the other allele (75). Conversely, mutations in *SF3B1* are associated with intermediate prognosis and risk of metastasis, while aberrations in *EIF1AX* signifies good prognosis and correlates with low metastatic risk (82). SCNAs associated with poor prognosis include monosomy 3, amplification of chromosome 8q and deletion of chromosomes 1p, 8p or 16q (68–72). Monosomy 3 occurs in just under half of UM cases and is associated with increased metastasis risk and reduced disease-free survival, and these deleterious correlations are further exacerbated by concomitant chromosome 8q gains (89). UM patients with monosomy 3 or chromosome 8q gains alone have a 5-year mortality rate of 40% and 31% respectively, rising to 66% for those with both monosomy 3 and chromosome 8q gains. Loss of chromosome 1p or 16q is associated with poor prognosis, while having diploid chromosome 3 or chromosome 6p gain correlates with lower metastatic risk and good prognosis (70, 90, 91). Accurate assessment of patient metastatic risk will empower patients to make life-changing decisions (92), and inform clinicians on patient care for example in guiding surveillance frequencies. Advancements in ctDNA detection methods to accurately assess these genetic aberrations and validation through clinical studies will be crucial prior to implementation into standard-of-care.

Recent clinical data indicate that ctDNA-based approaches may be utilized in stratifying UM patients for targeted therapies, monitoring therapeutic response, and predicting treatment resistance. In a phase 1 clinical trial of 17 metastatic UM patients treated with PKC inhibitor-based therapies, ctDNA accurately predicted patients with clinical benefits to PKC inhibitors and helped detect disease progression (25). Furthermore, NGS sequencing of ctDNA provided information of potential resistance mechanisms such as identifying loss of function mutations in *TP53* prior to radiological evidence of disease progression. In another study where pooled analysis was performed in patients with UM, NSCLC and CRC treated with nivolumab or pembrolizumab monotherapy, patients with ctDNA that became undetectable post treatment correlated with lasting treatment response (93). Conversely, patients with detectable ctDNA levels after treatment had reduced progression-free survival (HR = 10.2, 95% CI 2.5-4.1, P < 0.001) and overall survival (HR = 15, 95% CI 2.5-94.9, P = 0.004), indicating that ctDNA can be a marker of treatment failure and may inform early switching of treatment regimes. On the other hand, ctDNA-based approaches may also help with informing adjuvant therapy. In a trial involving stage II CRC patients, ctDNA-based approaches reduced adjuvant treatment use while not compromising patient outcome including survival (94). In that study, patients were randomized into two groups, and were given adjuvant treatment guided either by ctDNA-based methods or clinicopathological features. Patients negative for ctDNA were identified as low risk and not given adjuvant therapy, resulting in almost half the proportion of patients receiving adjuvant in the ctDNA-guided group compared to the group where adjuvant therapy decisions were guided by clinicopathological features. Strikingly, the two groups had comparable two-year recurrence free survival at 93.5% and 92.4% for the ctDNA-guided and clinicopathological-guided groups respectively, highlighting that ctDNA-based approach is non-inferior to standard management. Whether ctDNA-based approaches may inform adjuvant treatment in UM remains to be explored in future studies.

In patients with known metastatic UM, ctDNA levels correlated with disease burden (P = 0.002), and increasing ctDNA was found to precede evidence of radiological progression with a lead-time of 4-10 weeks (25), suggesting ctDNA as a potential useful marker to assess treatment response. In fact, the need of additional methods to monitor treatment response in addition to existing radiological criteria such as Response Evaluation Criteria in Solid Tumors (RECIST) is evident in recent phase 2 and phase 3 trials treating treatment-refractory metastatic UM patients with tebentafusp (95, 96). In the phase 2 study, tebentafusp treatment resulted in a 1-year overall survival of 62% (95% CI 53-70) with a median survival of 16.8 months (95% CI 12.9-21.3), compared to a historical 37% overall survival rate and median overall survival of 7.8 months (95). The phase 3 trial confirmed these findings with the tebentafusp group achieving a 1-year overall survival of 73% compared to 59% in the control group (HR for death = 0.51, 95% CI 0.37 to 0.71) (96). Importantly, these studies found that the benefit of tebentafusp treatment is beyond those observed using traditional radiological criteria, where only 5% (95% CI 2-10) and 9% (95% CI 6-13) of patients showed an objective response based on RECIST v1.1 in the phase 2 and phase 3 studies respectively, despite the marked improvement in survival (95, 96). Importantly, ctDNA was found to be a robust early indicator of clinical benefit to tebentafusp. Early reduction in ctDNA upon tebentafusp treatment was associated with improved overall survival even in patients with radiological progression of disease, and the degree of ctDNA reduction further correlated with more prolonged survival (8, 95). One potential explanation is that tebentafusp being a T cell redirection-based immunotherapy, may result in pseudoprogression radiologically as a consequence of increased immune cell infiltration into tumors, while a reduction in ctDNA may signify treatment response and cancer clearance. Indeed, in other cancer types such as cutaneous melanoma, ctDNA have also been found to reliably differentiate pseudoprogression from true progression in patients treated with immunotherapy (97). Utilizing ctDNA-based approach, though limited to only those with detectable ctDNA, may therefore be more suitable to radiological methods in monitoring early immunotherapy efficacy, and can potentially help guide clinical decision making on identifying responding patients to continue therapy while switching to alternatives for those unlikely to respond.

## Future directions

Prior to the advent of tebentafusp, systemic therapies such as immune checkpoint inhibitors showed limited efficacy in prolonging survival in metastatic UM, yet studies consistently demonstrate that the majority of UM patients wish to know regarding prognostic information on metastatic risk (92). Fast forward to now, early prediction and diagnosis of metastasis are more important than ever given the recent, unprecedented success of tebentafusp in treating metastatic UM, which improved median overall survival to 21.6 months for those that received tebentafusp from 16.9 months in controls (HR for death = 0.68, 95% CI 0.54 to 0.87) in a phase 3 randomized-controlled trial (8). Three-year survival was 27% and 18% in the tebentafusp group and control group respectively. In a recent meta-analysis on systemic treatments for metastatic UM, tebentafusp was found to result in the highest median OS (22.4 months, 95% CI 19.9-29.6) superior to combined immune checkpoint inhibitors (median OS = 15.7 months, 95% CI 14.4-17.9) and chemotherapy (median OS = 9.95 months, 95% CI 8.9-11.2) (98). It is therefore crucial to develop strategies to detect metastasis early in UM patients, which will provide lead time and allow timely intervention with tebentafusp with the potential to further improve patient survival.

On this notion and with exciting prospects, ctDNA-based approaches may serve as a potential suitable strategy to monitor disease recurrence after resolution of primary UM post definitive treatment, and assess for MRD which may predict or indicate metastasis. In one study, ctDNA signal was detected 2-10 months before clinical diagnosis of metastasis (26), potentially providing valuable lead time for early intervention. Shorter intervals between ctDNA liquid biopsies may provide an even longer lead time, and may be of particular benefit in UM patients deemed to be high-risk of metastasis. Detection rate of ctDNA in metastatic UM patients ranges from 35% to 94% depending on the patient cohort and the laboratory technique used (25, 26, 99, 100), and given the relatively low rate of ctDNA detection in primary UM, the presence of detectable ctDNA itself may favor metastatic disease and alert clinicians for further detailed investigations. Indeed in other cancer types such as prostate cancer, detection of ctDNA have also been associated with the presence of metastasis (101), and can facilitate early detection of breast cancer recurrence prior to imaging as shown in a recent case series (102). Further studies in the form of clinical trials are required to explore and validate these clinical utilities in the context of UM.

Taking it a step further, an exciting phase 2 multi-center trial currently open for recruitment named TebeMRD (EudraCT number: 2019-003946-34) aims to address the safety and efficacy of tebentafusp in melanoma patients with MRD prior to overt clinical relapse or metastasis. The study includes both cutaneous melanoma and UM patients who have undergone conventional definitive treatment with no residual disease detectable on imaging at enrolment, and addresses the important question of utilizing ctDNA for early detection of cancer molecular relapse. Furthermore, TebeMRD explores the potential to therapeutically intervene at the point of detecting MRD and provide insight on whether this may lead to clinical benefit, as opposed to careful surveillance for clinical relapse. The rationale behind this approach is based on evidence in metastatic UM patients where changes in ctDNA levels preceded radiological progression by 4-10 weeks (25) and are a robust early indicator of tebentafusp clinical efficacy (95), and that a marked improvement in patient survival was observed in clinical trials despite only a very a small proportion of patients demonstrating objective response to tebentafusp based on RECIST criteria (8, 95). Whether early intervention at the point of detecting MRD prior to clinical relapse or metastasis can achieve meaningful benefits is an exciting avenue to explore with the potential to improve patient prognosis and outcome.

**Figure 1:**
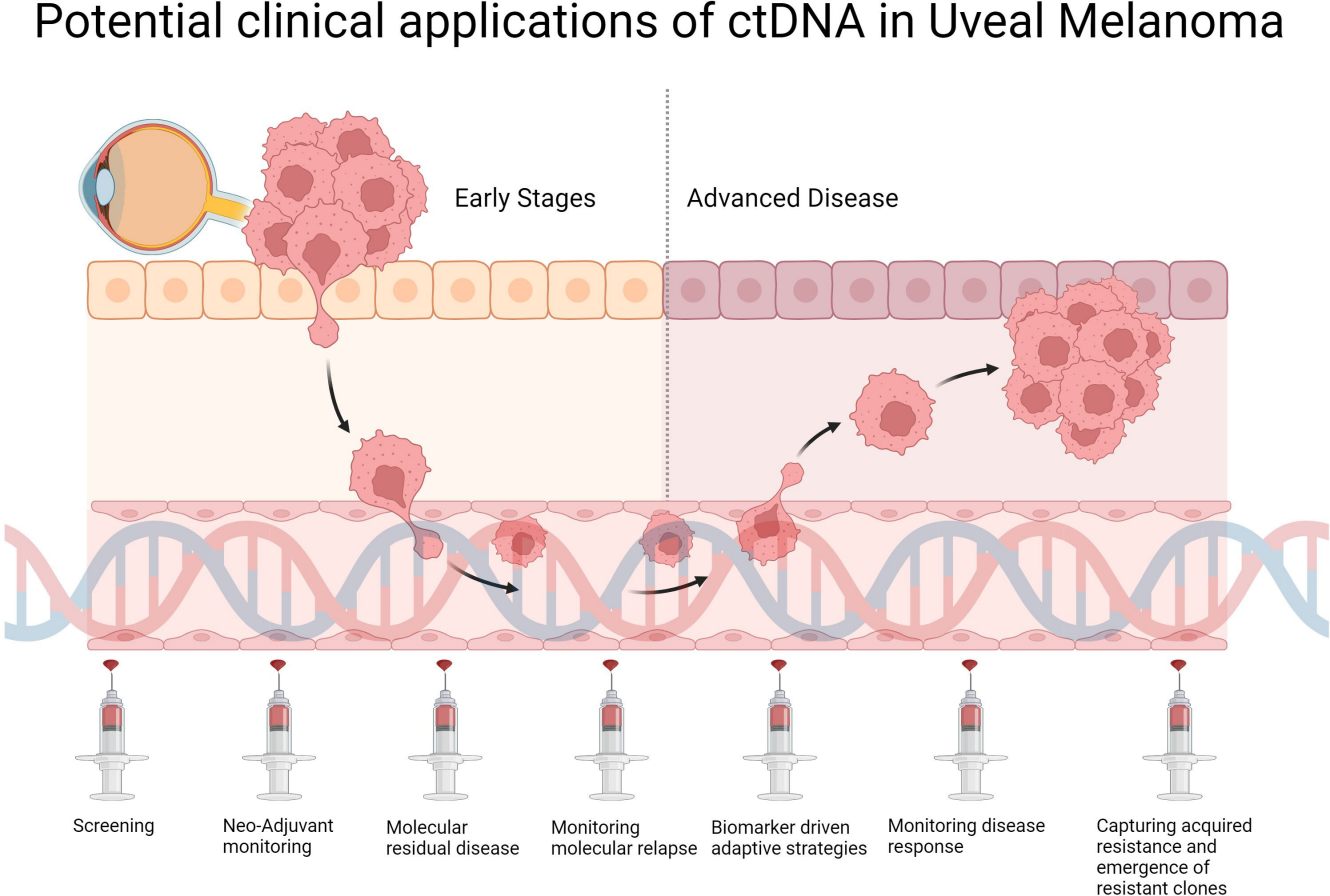
Potential clinical applications and integration of ctDNA into future clinical workflow of UM management.

Going forwards, implementation of liquid biopsies into routine clinical practice for UM holds exciting potential (**Figure 1**), however several challenges must be overcome prior to integrating ctDNA-based approaches into the existing clinical workflow. Further technological advances are required to lower the limit of detection to allow robust detection of early relapse and MRD. In cases where tumor-informed approaches are adopted for ctDNA analyses, care must be taken as synchronous or metachronous cancers may be missed as highlighted in a recent CRC case study (103). Standardized methods to detect and assess ctDNA are required across laboratories to achieve reliable, comparable and reproducible results. Factors that may affect ctDNA detection sensitivity, specificity and result concordance include the timings of sample collection, the sample collection procedure used, the handling time prior to analysis, the storage methods and conditions, the mutations assessed, and the different laboratory techniques used for sample processing, library preparation and ctDNA detection. Efforts to standardize these processes and by organizations such as the International Liquid Biopsy Standardization Alliance, European Liquid Biopsy Society and the National Cancer Institute, and the development of validated reference materials, will be crucial to facilitate ctDNA-based approaches into the clinic for patient care (104–106). Cost-effectiveness of ctDNA-based strategies compared to traditional methods is another key parameter that needs to be assessed in future studies. A recent study modelled the cost-effectiveness of ctDNA in aiding selection for adjuvant treatment for CRC patients in the Netherlands, and predicted that combining ctDNA with existing traditional strategies would be cost effective if test costs can be lowered to below €1500, or if ctDNA status can effectively predict therapeutic response in these patients (107). Another recent study projected that ctDNA testing in the USA, also on stage II CRC patients, is likely to be cost-effective for both commercial and Medicare Advantage patients (108). Advances in ctDNA detection technologies with standardized procedures will improve the accuracy and lower the cost of ctDNA testing, and should be validated in clinical trials assessing for safety, efficacy and cost-effectiveness in clinical patient care.

## Conclusions

Growing strategies to harness ctDNA-based approaches in solid cancer patient care show great promise with a wide range of potential clinical utilities including diagnosis of initial cancer or relapse, disease monitoring, prognostication, early identification of metastasis, assess treatment response, and detection for MRD thereby facilitating early intervention. UM as a cancer type is particularly amenable to ctDNA-based strategies, due to the relatively well-characterized mutational landscape and the primary anatomical site making traditional biopsies less favorable. With tebentafusp showing improved survival in metastatic UM patients, early detection of disease relapse and metastasis is more important than ever. This also opens avenues for potentially utilizing tebentafusp in adjuvant and MRD settings, in conjunction with ctDNA-based approaches which may identify patients most likely to benefit from adjuvant or detect MRD respectively. Nevertheless, ctDNA in UM is largely limited to research settings currently. Going forwards, technological advances and strategies to improve sensitivity of ctDNA detection, along with standardization of techniques across laboratories and robust clinical validation will be crucial prior to implementation into routine clinical practice.
